# Acute Phase Response-driven Hepatic Niche Remodeling Promotes Fibrosis Resolution After Alcohol Cessation

**DOI:** 10.1016/j.jcmgh.2025.101689

**Published:** 2025-11-29

**Authors:** Michael Schonfeld, Kruti Nataraj, Samson Mah, Wei Zhong, Steven A. Weinman, Irina Tikhanovich

**Affiliations:** Department of Internal Medicine, University of Kansas Medical Center, Kansas City, Kansas

**Keywords:** HDL, KDM5 Demethylases, Serum Amyloid A, Single-cell Sequencing, Spatial Transcriptomics

## Abstract

**Background & Aims:**

Abstinence is beneficial for patients with alcohol-associated liver disease (ALD), but disease resolution after alcohol cessation occurs slowly and only in a subset of patients. We aimed to study the mechanisms of ALD resolution using spatial transcriptomics.

**Methods:**

Mice were fed Western diet with 20% alcohol in the drinking water for 20 weeks followed by chow diet with plain water for 4 weeks. Livers were analyzed by 1000-plex CosMx spatial transcriptomics assay (Nanostrings). To assess the role of serum amyloid A (SAA), mice were treated with recombinant SAA or SAA-rich high-density lipoprotein (HDL).

**Results:**

Using a mouse model of ALD after alcohol cessation we performed spatial transcriptomics and identified a discrete multicellular fibrogenic and fibrolytic niches. Fibrolytic niches contained a unique subpopulation of hepatocytes that express SAA. SAA expression correlated with fibrolytic genes in mice after alcohol cessation and in human liver samples. In vitro analysis confirmed that *Saa1/2*^high^ hepatocytes induced matrix metalloproteinase and lysosomal enzyme (*Ctsd, Psap*) gene expression in liver macrophages in an SAA and FPR2-dependent way. Moreover, after alcohol cessation, SAA was enriched on circulating HDL and the SAA pro-resolving function required SR-BI-mediated HDL uptake by macrophages. In vivo recombinant SAA or SAA-enriched HDL promoted fibrosis resolution after alcohol cessation in mice. SAA expression itself was mediated by IL-22R signaling in hepatocytes regulated by KDM5B demethylase and C/EBPβ. Hepatocyte-specific *Kdm5b* or *Cebpb* knockout promoted *Il22a1* expression, *Saa1/2* upregulation and collagen remodeling, facilitating fibrosis resolution after alcohol cessation.

**Conclusions:**

Acute phase response activation after alcohol cessation triggers intrahepatic cell-cell communication changes for efficient fibrosis resolution.

## Graphical abstract

**Figure:**
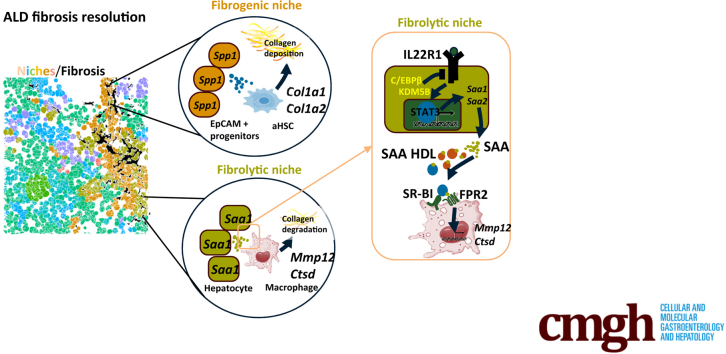

**Summary**

Serum amyloid A (SAA) promotes fibrosis resolution after alcohol cessation in alcohol-associated liver disease. SAA mediates fibrolytic shift in liver macrophages during resolution in FPR2 and SR-BI-dependent way. KDM5B and C/EBPβ suppress SAA activation via downregulation of upstream interleukin-22 receptor.

**What You Need to Know**

**Background**

Abstinence is beneficial for patients with alcohol-associated liver disease (ALD), but disease resolution after alcohol cessation occurs slowly and only in a subset of patients.

**Impact**

This study uncovers a mechanism involved in ALD disease resolution. We found that acute phase response (APR) after alcohol cessation triggers intrahepatic cell-cell communication changes that promote fibrosis regression.

**Future Directions**

In the future, we will investigate the mechanism involved in APR activation after alcohol cessation and will study APR in a prospective cohort of patients with ALD who stop drinking.

Alcohol-associated liver disease (ALD) encompasses a spectrum of disorders that commonly progress from steatosis to steatohepatitis with fibrosis and ultimately to cirrhosis. Cirrhosis is the ninth leading cause of death in the United States and about 35% to 50% of cirrhosis deaths are alcohol-related.^1^ Cessation of drinking is critically important for survival after development of ALD, but unfortunately recovery of liver function after alcohol cessation is slow and, in many cases, does not occur.^2^ The reasons for poor disease recovery in ALD are poorly understood.

In ALD, multiple liver cell types are impacted by alcohol exposure; however, it is not clear if effects on individual cells are direct effects of alcohol, or a response to alcohol-induced signals from other cells. Advances in technologies such as single-cell sequencing and spatial transcriptomics have revealed new details regarding the complexity of hepatic cell types^3^ and have highlighted the importance of cell-cell communication via secreted mediators such as peptides, hormones, and cytokines in disease development and progression.^4^ Recent studies suggest that hepatocytes play the central role in driving ALD, and they generate signals that influence the phenotype of nonparenchymal cells (NPCs) in the liver both during disease development^5^ and during the course of disease resolution.^6^

In this study, we have examined the role of the hepatocyte acute phase response (APR) in fibrosis recovery from ALD. APR is the rapid reprogramming of gene expression and metabolism in response to inflammatory cytokine signaling to limit the spread of infection and to promote tissue repair.^7^ Hepatocyte-derived acute phase proteins (APPs) play a central role as mediators of the inflammatory processes, enhancing phagocytosis, and helping clear the products of inflammation. Classic APR signaling is an early defense mechanism aimed at restoring tissue homeostasis. However, in chronic inflammation, sustained production of certain APPs can be deleterious and promote metabolic dysfunction,^8^ but it may also play a role in recovery from chronic inflammation. The role of APR in chronic disease resolution has not yet been studied.

Serum amyloid A (SAA) is an important APP that is involved in tissue repair after acute injury.^9^, ^10^, ^11^, ^12^, ^13^, ^14^ SAA is known to induce matrix metalloproteinases (MMPs) and tissue remodeling both after acute injury and in chronic conditions.^12^^,^^15^^,^^16^ SAA can stimulate cholesterol efflux,^8^^,^^17^^,^^18^ which we recently identified to be crucial for ALD resolution.^19^

In this work, we identified a subset of SAA-expressing hepatocytes as a key component of a fibrolytic niche within the liver that is associated with fibrosis resolution after alcohol cessation. We found that SAA and SAA-enriched high-density lipoprotein (HDL) stimulated niche remodeling to promote fibrolytic properties in liver macrophages in an FPR2 and SR-BI dependent way. Taken together, these observations indicate that activation of APR signaling in hepatocytes alters the phenotype of NPCs and stimulates pro-resolving changes required for collagen degradation and fibrosis resolution.

## Results

### Spatial Transcriptomics Defines Functionally Distinct Niches in the Liver After Alcohol Cessation

**Figure 1:**
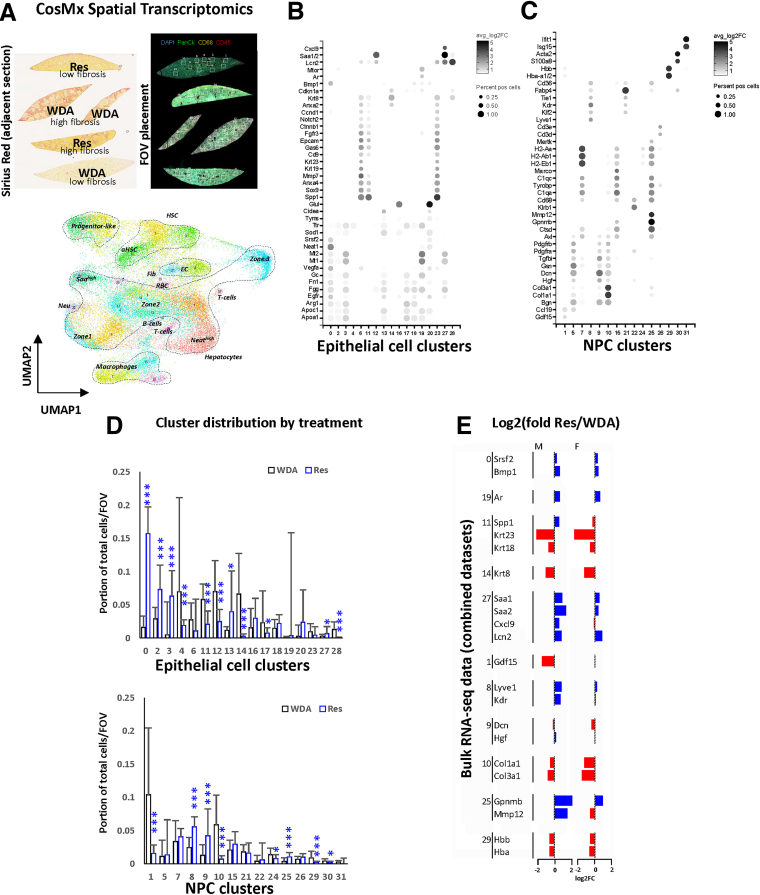
**Alcohol cessation promotes transcriptomic changes in hepatocytes and non-parenchymal cell clusters.** Mice were fed ad libitum WD with 20% alcohol in the drinking water for 20 weeks (WDA). For disease resolution mice were placed then on chow diet with plain water for 4 weeks (Res). Spatial transcriptomics analysis of livers from these mice (n = 2 per group, males) using 1000-plex assay from Nanostrings using CosMx platform. Cell boundary was assigned based on nuclear and membrane staining. Cells were analyzed using Seurat v5 package. (*A*) *Top*. Sirius red staining of the section adjacent to section used for transcriptional profiling. *Bottom.* UMAP plot of cell clusters. (*B* and *C*) Gene expression of genes enriched in individual clusters. (*D*) Cell abundance for each cluster across 45 FOVs. Average proportion for each cluster in WDA and Res FOVs (n = 22–23 per condition). ∗*Padj* < .01; ∗∗∗*Padj* < .0001. (*E*) Fold change for indicated gene transcripts between Res and WDA samples in males (M) and females (F).

We previously reported that fibrosis resolution after alcohol cessation is impaired due to epigenetic changes induced by KDM5B demethylase and continuous activation of the C/EBPβ transcription factor in hepatocytes.^19^^,^^20^ However, how hepatocyte driven cell-cell communication controls the fibrosis resolution process was not clear. To assess cell-cell communication in the liver during ALD development and resolution, we explored spatial gene expression using a 1000-plex hybridization assay as part of the NanoString CosMx platform (Figure 1). Mice were fed a high-fat Western diet (WD) with 20% alcohol in the drinking water (WDA model: average, 20 g/kg/day alcohol intake; 33% daily calories from fat^21^) for 20 weeks and then placed on chow diet with plain water for 4 weeks (Resolution). Liver fibrosis was assessed by Sirius Red staining of an adjacent section (Figure 1*A*).

Using spatial transcriptomics in combination with membrane and nuclear staining to define cell boundaries, we assigned detected transcripts to individual cells and performed cell clustering using the Seurat 5.0 package (Figure 1*A*). We were able to identify 32 clusters (Supplementary Table 1; Figure 1*B–C*) including 17 epithelial cell clusters (3 *Epcam* ^high^ *Spp1* ^high^ progenitor-like cells and 14 hepatocyte clusters) (Figure 1*B*), and 15 NPC clusters (3 macrophage clusters, 3 hepatic stellate cell [HSC] clusters, 2 clusters of endothelial cells, T-cell, B-cell, neutrophil clusters, etc) (Figure 1*C*). Despite defects in cell segmentation, several relevant cell populations were detected with high confidence. For example, pro-resolving macrophages (cluster 25, *Mmp12* ^high^ *Ctsd* ^high^) that can promote collagen degradation because of high expression of MMPs^22^ and lysosomal genes such as *Psap* (prosaposin) and *Ctsd* (cathepsin D, involved in pro-resolving shift in liver macrophages^23^). Another example is activated HSCs (aHSCs, cluster 10, *Col1a1*^high^), which are involved in collagen deposition (Figure 1*C*). We next examined changes in cell type abundance between WDA and Resolution samples (Figure 1*D*). We found dramatic differences in cell abundance for both epithelial and NPC clusters after alcohol cessation. Specifically, we found a 6-fold increase in *Neat1* ^high^ *Srsf2* ^high^ cells (cluster 0) and 2-fold increase in *Saa1/2* ^high^ *Crp* ^high^ *Cxcl9* ^high^ hepatocytes (cluster 27), but a 2-fold decrease in *Epcam* ^high^ *Spp1* ^high^ progenitor-like cells (clusters 6, 11, 23), and a 10-fold decrease in *Krt8* ^high^ (cluster 14) cells. Among NPC populations, we found a 2-fold increase in *Lyve1* ^high^ *Kdr* ^high^ endothelial cells (cluster 8), *Mmp12* ^high^ macrophages (cluster 25), and a 5-fold decrease in *Tyms* ^high^ *Gdf15* ^high^ cells (cluster 1) and a 6-fold decrease in *Col1a1*^high^ aHSC (cluster 10).

To validate our findings, we examined whole liver mRNA changes from RNA sequencing (RNA-seq) analysis of WDA and 4-week resolution livers (GSE244240, GSE276692). We found that in both male and female livers we observed corresponding changes in whole liver mRNA for multiple specific gene markers for each of the changing clusters (Figure 1*E*).

**Figure 2:**
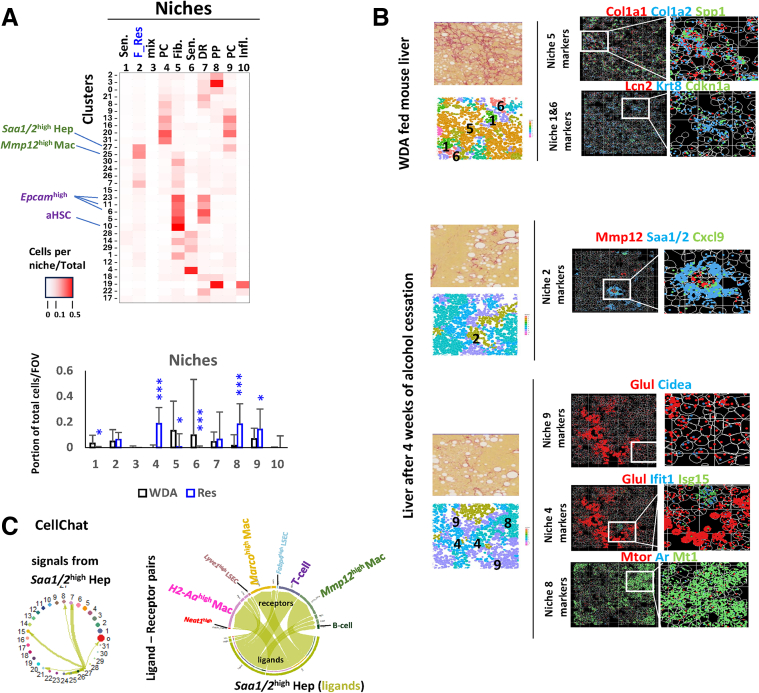
**Spatial transcriptomics identifies functional niches in the liver during ALD resolution.** (*A*) Niche analysis showing cell cluster proportion in each niche and niche abundance changes in WDA and Res FOVs. (*B*) Example FOV showing region with indicated niches. *Left top.* Sirius red staining. *Left bottom.* Cell segmentation showing niches. *Right.* Identified mRNA positions of key transcripts. (*C*) CellChat analysis of cell-cell communication identified L-R interactions between *Saa1/2* high hepatocytes and NPC clusters.

We next used k-means clustering to group cells into spatial niches (Figure 2*A* and *B*). In this context, niches represent multicellular local groupings that occur repeatedly throughout the specimen. We detected 2 niches representing the pericentral zone (niches 4 and 9) and 1 niche for the periportal zone (niche 8), 2 niches enriched in senescent cells (niche 1 and 6), and a niche enriched in progenitor-like cells or ductular reaction (niche 7). In addition, we identified 1 fibrogenic niche containing a majority of the *Col1a1*^high^ stellate cells (niche 5) and 1 fibrolytic niche enriched in *Mmp12* ^high^ macrophages (niche 2). The fibrogenic niche was enriched with *Epcam* ^high^ *Spp1* ^high^ progenitors (clusters 6, 11, 23), *Tyms* ^high^ *Gdf15* ^high^ (cluster 1), and *Krt8* ^high^ (cluster 14) cells. As we reported previously, collagen synthesis was absent in livers after alcohol cessation,^19^ which correlated with the absence of niche 5 in livers at 4 weeks resolution (Figure 2*A*). In contrast, we observed an increase in niches 4, 8, and 9 (Figure 2*A*) enriched for well-differentiated hepatocytes (*Ttr* ^high^) and liver sinusoidal endothelial cells (LSECs) (*Lyve1* ^high^ *Kdr* ^high^).

The fibrolytic niche (niche 2) was enriched for *Saa1/2* ^high^ *Crp* ^high^ *Cxcl9* ^high^ hepatocytes (cluster 27) (Figure 2*A, B*; Supplementary Table 1). Interestingly, the fibrolytic niche proportion did not significantly increase after alcohol cessation, suggesting defective fibrosis resolution in wild-type (WT) mice as reported before.^19^ Examining transcript distribution for *Mmp12, Saa1/2,* and *Cxcl9* we found close association between *Saa1/2* ^high^ *Cxcl9* ^high^ hepatocytes and *Mmp12* ^high^ macrophages (Figure 2*B*), suggesting possible communication between these cell types. We then used the CellChat tool^24^ to examine cell-cell communication (Figure 2*C*). We identified several ligand-receptor (L-R) interactions between *Saa1/2* ^high^ hepatocytes and *Mmp12* ^high^ macrophages as well as *Saa1/2* ^high^ hepatocytes and other NPC clusters such as T-cells, LSECs, and other macrophage clusters, but no HSC clusters (clusters 5, 9, 10), suggesting that *Saa1/2* ^high^ hepatocytes modulate fibrolytic but not fibrogenic niche function.

### APR Signaling is Induced During Recovery and Correlates With Hepatocyte Ability to Induce Macrophage Pro-resolving Phenotype

**Figure 3:**
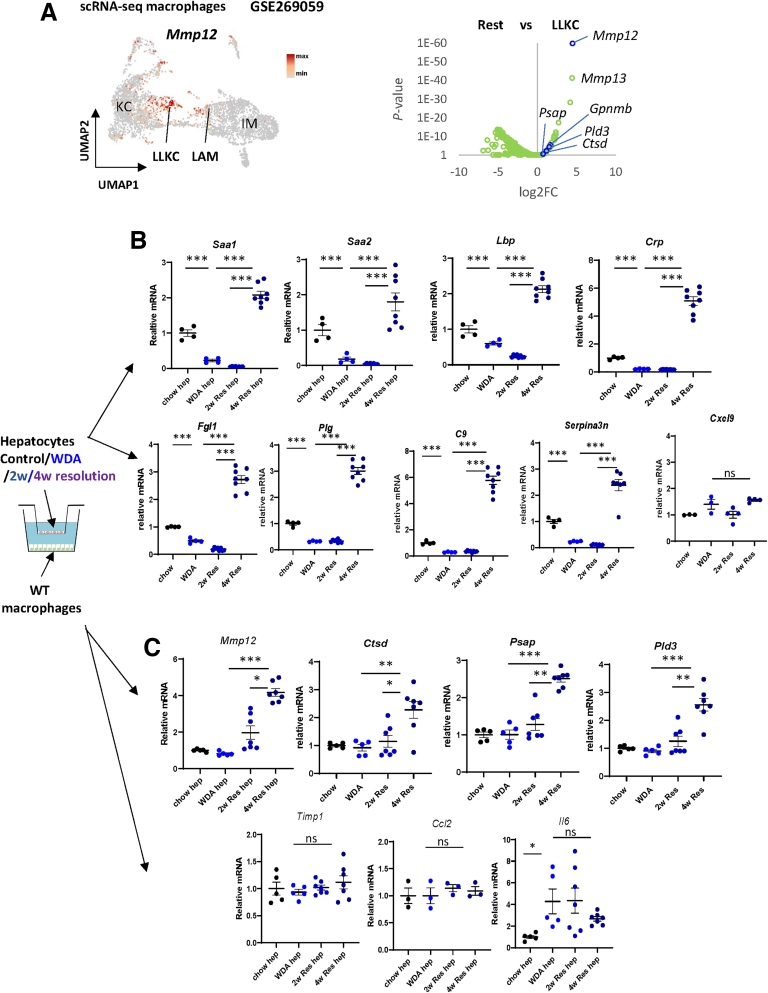
**Hepatocytes drive pro-resolving changes in liver macrophages during ALD resolution.** (*A*) *Left. Mmp12* gene expression in liver macrophages in mice fed WDA (GSE269059). *Right.* Cluster 25 enriched genes (*blue*) mapped to differential gene expression between LLKC (*Mmp12* expressing macrophages) and other macrophages id scRNA-seq dataset. (*B*) Hepatocytes were isolated from chow-fed mice, mice fed WDA for 20 weeks, or mice fed WDA for 20 weeks and placed on chow diet with water for 2 or 4 weeks (2w Res, 4w Res). *Top.* Relative gene expression in hepatocytes. *Bottom.* Hepatocytes were co-cultured in a transwell assay with liver macrophages isolated from chow-fed mice. Relative gene expression in macrophages. N = 4–8 per group. ∗*P* < .05; ∗∗*P* < .01; ∗∗∗*P* < .001.

Cluster 25 enriched genes (*Mmp12, Gpnmb, Psap, Ctsd, Pld3*) are highly enriched in previously described lipid-associated macrophage (LAM)-like Kupffer cell (KC) (LLKC) subset of liver macrophages that we reported previously^22^ (Figure 3*A*). These macrophages represent a subset of monocyte-derived macrophages with high MMP and lysosomal gene expression and are likely involved in collagen degradation.^22^^,^^23^^,^^25^ However, signals required for the emergence of this subset are not clear. Previous studies reported that acute phase proteins, such as SAA may induce MMP gene expression.^16^

SAA and C-reactive protein (CRP) are acute phase proteins involved in inflammatory responses and wound healing. We further examined APR gene expression in hepatocytes after alcohol cessation. Mice were fed WDA for 20 weeks and then placed on chow diet with plain water for 2 or 4 weeks (Figure 3*B*). We found that in isolated hepatocytes, APR genes were induced after 4 weeks of alcohol cessation compared with WDA end of treatment (Figure 3*B*). In contrast, *Cxcl9* gene expression was not changed (Figure 3*B*). We next used these hepatocytes in a co-culture with liver macrophages isolated from chow-fed untreated mice. We found that hepatocytes from 4-week resolution were able to induce pro-resolving phenotype in macrophages corresponding to gene expression in cluster 25 (*Mmp12* ^high^*, Ctsd* ^high^) (Figure 3*C*). In contrast, proinflammatory genes (*Ccl2, Il6*) were not affected in macrophages (Figure 3*C*).

**Figure 4:**
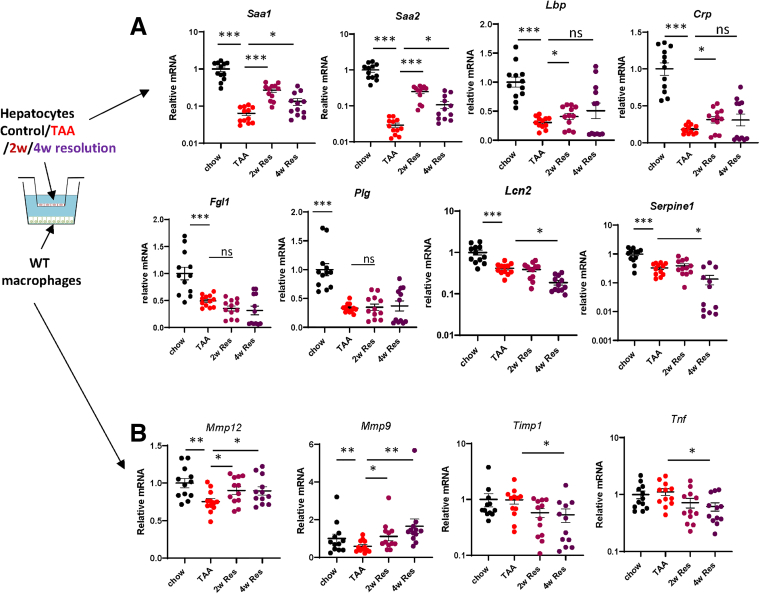
**Hepatocytes drive pro-resolving changes in liver macrophages during TAA resolution.** Mice were given TAA in the drinking water for 10 weeks or given TAA and returned to plain water for 2 or 4 weeks (2w Res, 4w Res). N = 12 per group. ∗*P* < .05; ∗∗*P* < .01; ∗∗∗*P* < .001. Hepatocytes were co-cultured with macrophages from chow-fed mice. Gene expression in hepatocytes (*A*) and macrophages (*B*).

We tested whether these changes exist during liver disease resolution in another model of liver fibrosis. Mice were treated with thioacetamide (TAA) in the drinking water for 10 weeks and let recover for 2 or 4 weeks (Figure 4*A*). We found that although hepatocyte APR was suppressed by the disease model itself, like in the WDA model, hepatocyte APR was induced after TAA withdrawal. However, *Saa1/2* induction was more prominent at 2 weeks resolution timepoint compared with TAA end of treatment (Figure 4*A*). Using a co-culture system with liver macrophages, we confirmed that APR induction in hepatocytes correlated with their ability to induce pro-resolving gene expression changes in liver macrophages (ie, more MMP and less tissue inhibitor of metalloproteinases [TIMP] gene expression) (Figure 4*B*), although this effect is less prominent in the TAA model as compared with WDA.

Taken together, these data suggest that hepatocytes upregulate APR and specifically SAA after alcohol cessation, which correlates with their ability to promote pro-resolving macrophage phenotype.

### SAA Expression Correlates With Improved Fibrosis Resolution in Mice

**Figure 5:**
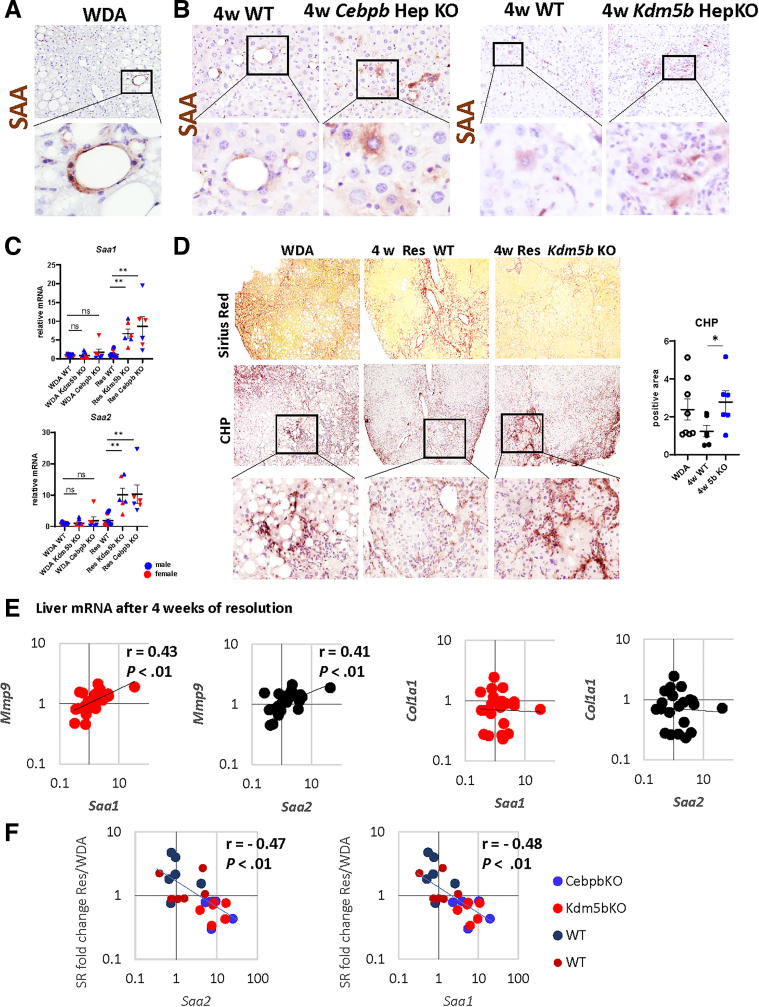
**Hepatocyte SAA positively correlates with increased fibrosis resolution in mice.** (*A–B*) SAA protein expression in WDA-fed mice or in WT, *Kdm5b* KO, or *Cebpb* KO mice at 4 weeks after alcohol cessation. (*C*) *Saa1* and *Saa2* gene expression in WT, *Kdm5b* KO, or *Cebpb* KO mice fed WDA or at 4 weeks after alcohol cessation in males (*blue*) and females (*red*). N = 3. ∗∗*P* < .01. (*D*) Sirius Red staining and CHP staining in WDA-fed mice or in WT, *Kdm5b* KO mice at 4 weeks after alcohol cessation. *Right.* CHP-positive area. N = 6–8. ∗*P* < .05. (*E*) *Saa1* and *Saa2* gene expression in WT, *Kdm5b* KO, or *Cebpb* KO mice at 4 weeks after alcohol cessation correlates with *Mmp9* gene expression in these mice but not *Col1a1* gene expression. (*F*) Correlation between *Saa1/2* and Sirius Red staining fold change (4-week resolution/WDA biopsy) in WT and KO mice.

Previously, we reported fibrosis resolution after alcohol cessation is enhanced in hepatocyte-specific *Kdm5b* knockout (KO)^19^ and hepatocyte specific *Cebpb* KO^20^ mice. We evaluated SAA expression and protein abundance in these mice at 4 weeks after alcohol cessation (Figure 5). SAA levels were very low in WDA-fed mice (Figure 5*A*); protein was observed mainly within crown-like structures. After 4 weeks of resolution, we found that, in WT mice, hepatocyte SAA levels were slightly elevated in agreement with mRNA changes (Figure 5*B*). In contrast, both *Kdm5b* KO and *Cebpb* KO mice had greatly elevated SAA protein staining in hepatocytes (Figure 5*B*) and *Saa1, Saa2* mRNA levels at 4 weeks after alcohol cessation (Figure 5*C*). We next evaluated whether the SAA increase correlated with niche remodeling during the recovery phase in these mice. We previously reported that these KO mice had improved fibrosis resolution evident by reduced Sirius Red staining. To evaluate extracellular matrix (ECM) remodeling, we used a collagen hybridizing peptide (CHP) to mark sites of collagen remodeling/degradation. We found that reduced fibrosis in *Kdm5b* KO mice correlated with increased ECM remodeling (Figure 5*D*). We next assessed if SAA upregulation correlated with fibrolytic gene expression (Figure 5*E*). We found that *Saa1* and *Saa2* positively correlated with *Mmp9* gene expression in the livers of WT and KO mice after 4 weeks of alcohol cessation. In contrast, *Saa1* and *Saa2* did not correlate with profibrotic genes such as *Col1a1*. Moreover, *Saa1/2* strongly correlated with a decrease in fibrosis in these mice measured by fold change (FC) in Sirius red staining at 4 weeks post alcohol relative to WDA biopsy controls for each mouse (Figure 5*F*).

Taken together, these data suggest that increased fibrosis resolution in *Kdm5b* and *Cebpb* KO mice correlates with SAA upregulation.

### SAA-expressing Hepatocytes Correlated With Pro-resolving Genes in Human ALD

**Figure 6:**
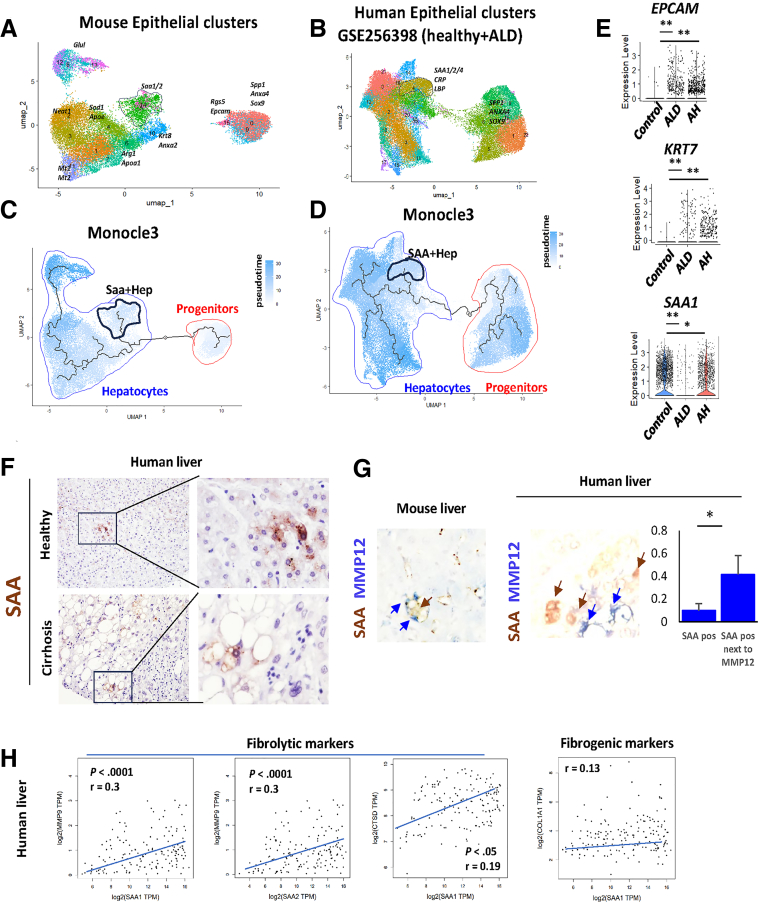
**SAA positively correlates with fibrolytic genes in human liver.** (*A–D*) Epithelial cell clusters from mouse spatial dataset were re-clustered (*A*) and analyzed with Monocle3 (*C*) to identify potential origin of *Saa1/2*^high^ cells (clusters 5 and 14). *Saa1/2*^high^ cells are outlined. Epithelial cell clusters from human snRNA-seq dataset (GSE256398, Healthy, ALD, and AH samples only) were re-clustered (*B*) and analyzed with Monocle3 (*D*) to identify potential origin of *SAA1/2/4*^high^ cells (cluster 4). *SAA1/2/4*^high^ cells are outlined. (*E*) Gene expression changes in human hepatocyte clusters in healthy controls, ALD cirrhosis, and AH. (*F*) SAA protein expression in healthy controls and ALD cirrhosis. (*G*) SAA and MMP12 protein co-localization in mice after 4 weeks of ALD resolution or in human liver cirrhosis samples. SAA-positive hepatocytes area in human liver compared with SAA-positive hepatocyte frequency next to MMP12-positive cell in human liver sections. ∗*P* < .05. (*H*) Correlation between *SAA1, SAA2* and *MMP9, CTSD* or *COL1A1* gene expression in human liver samples (TCGA control and GTEx).

We next assessed the relevance of SAA expressing hepatocytes in human ALD (Figure 6). We re-clustered epithelial cells from the mouse CosMx dataset (Supplementary Table 2). Clusters 5 and 14 showed enrichment for *Saa1/2* gene expression; clusters 0, 9, and 16 represented *Epcam* positive progenitors; clusters 8, 12, and 13 were pericentral hepatocytes; clusters 1 and 11 were periportal hepatocytes (Figure 6*A*). We next analyzed healthy human liver, ALD cirrhosis, and alcoholic hepatitis (AH) samples from a published small nuclear RNA (snRNA)-seq data set (GSE256398).^26^ We separated and re-clustered epithelial cell clusters (Figure 6*B*; Supplementary Table 3). Similar to mouse data, we identified APR cluster (cluster 4) that showed enrichment for *Saa1/2/4, CRP, LBP* and other APR genes; clusters 1, 5, 6, 8, and 9 that represented *EPCAM* positive progenitors; and other hepatocyte clusters (Figure 6*B*). We performed trajectory analysis in epithelial cell clusters from mice (Figure 6*C*) and humans (Figure 6*D*) using the Monocle3 package. We found similar trajectory patterns with *Saa1/2* ^high^ cells at the endpoint of a separate branch of a progenitor to hepatocyte differentiation trajectory, suggesting that SAA-expressing hepatocytes are present in both mice and humans and may have similar origin and function.

We further assessed changes in gene expression in hepatocytes from patients with ALD compared with healthy controls (Figure 6*E*), We found that in ALD samples, hepatocytes showed an increase in progenitor markers (*EPCAM, KRT7*) and a significant decrease in *SAA1*, suggesting that SAA correlates with disease development and progenitor markers accumulation. Using liver samples from the KUMC Liver Bank, we found that SAA protein levels are similarly reduced in liver cirrhosis samples compared with healthy controls (Figure 6*F*). Like in mouse livers, SAA in human cirrhosis localized in proximity to lipid droplets and crown-like structures.

We assessed whether SAA and MMP12 proteins co-localize in mouse and human liver samples (Figure 6*G*). We found that SAA was enriched in the proximity of MMP12-positive cells, suggesting that it might be involved in MMP induction. We further confirmed that *SAA1* and *SAA2* positively correlated with fibrolytic markers in human liver (*MMP9, CTSD*), but not with fibrogenic markers (*COL1A1*) (Figure 6*H*), suggesting strong association between SAA and the pro-resolving phenotype.

### SAA Expression in Hepatocytes Promotes the Macrophage Pro-resolving Phenotype

**Figure 7:**
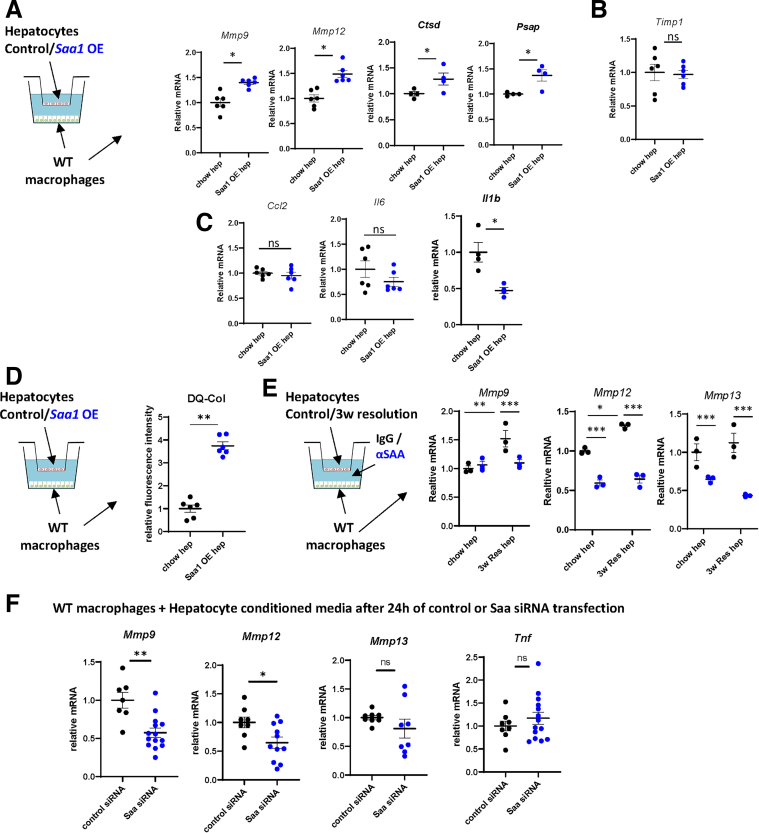
**Hepatocyte SAA drives pro-resolving changes in liver macrophages during ALD resolution.** (*A–D*) Hepatocytes were transfected with control vector or vector expressing mouse *Saa1* and were used in a co-culture system with liver macrophages from chow-fed mice. (*A–C*) Relative gene expression in macrophages. (*D*) Collagen degradation assay. N = 4–6. ∗*P* < .05; ∗∗*P* < .01. (*E*) Hepatocytes were isolated from chow-fed mice or mice fed WDA for 20 weeks and placed on chow diet with water for 3 weeks (3w Res). Hepatocyte-macrophage co-cultures were supplemented with neutralizing antibodies specific for SAA or IgG control (0.5 μg/mL). N = 3. ∗*P* < .05; ∗∗*P* < .01; ∗∗∗*P* < .001. (*F*) Hepatocytes were transfected with control siRNA or siRNA pool specific for mouse *Saa* After 24 hours, conditioned media were used to treat macrophages from chow-fed mice. N = 5–10. ∗*P* < .05; ∗∗*P* < .01.

To assess whether SAA upregulation in hepatocytes alone could induce pro-resolving changes in liver macrophages, we overexpressed the mouse *Saa1* gene in isolated hepatocytes and measured gene expression changes in co-cultured liver macrophages (Figure 7*A–D*). We found that *Saa1* overexpression promoted *Mmp9*, *Mmp12*, *Ctsd,* and *Psap* gene expression in macrophages (Figure 7*A*), but did not affect *Timp1* (Figure 7*B*) and reduced some of the proinflammatory gene expression (Figure 7*C*). Moreover, *Saa1* overexpression promoted macrophage collagen degradation ability (Figure 7*D*). We next tested whether SAA is required for hepatocytes isolated from alcohol cessation mice to induce pro-resolving changes in macrophages (Figure 7*E*). We found that antibody-mediated SAA depletion dramatically reduced *Mmp9*, *Mmp12*, and *Mmp13* gene expression in macrophages cultured with hepatocytes from 3-week resolution mice. Similarly, *Saa* knockdown in hepatocytes reduced macrophage *Mmp9*, *Mmp12* gene expression, but did not affect *Tnf* (Figure 7*F*). Taken together, these data suggest that SAA is necessary and sufficient to induce pro-resolving macrophage phenotype.

**Figure 8:**
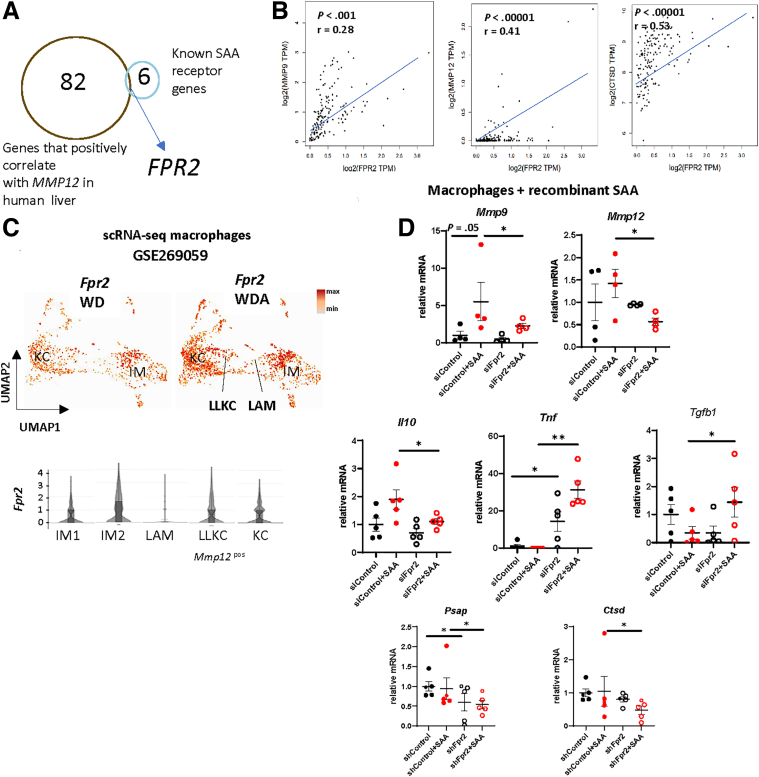
**Hepatocyte SAA drives pro-resolving changes in macrophages in FPR2-dependent way.** (*A*) Genes positively correlating with MMP12 in human liver (r > 0.7) overlap with known SAA receptor genes. (*B*) Correlation between *FPR2* and *MMP9, CTSD* or *MMP12* gene expression in human liver samples (TCGA control and GTEx). (*C*) *Fpr2* gene expression in liver macrophages in mice fed WD or WDA (GSE269059). Violin plots show gene expression in individual clusters from combined dataset. (*D*) Freshly isolated mouse macrophages were transfected with *Fpr2* siRNA or control siRNA and treated with recombinant SAA (5 μg/mL) for 24 hours. Relative gene expression in macrophages. N = 3–4. ∗*P* < .05; ∗∗*P* < .01.

There are several reported receptors for SAA that are involved in SAA downstream signaling activation. To assess which of the receptors are involved in MMP induction, we analyzed genes that positively correlate with MMP gene expression in human liver samples (Figure 8*A, B*). *FPR2* gene expression showed the strongest positive correlation with *MMP9*, *MMP12,* and *CTSD* in human samples, suggesting that it could be involved in SAA mediated pro-resolving changes. Using our previously reported scRNA-seq dataset, we found that *Fpr2* gene expression is induced in macrophages in alcohol-exposed livers compared with control (WDA vs WD alone) (Figure 8*C*). *Fpr2* expression was the highest in *Clec4e* positive IM (IM2) cells. *Fpr2* was significantly higher in LLKC (*Mmp12* ^pos^, *Ctsd* ^high^) compared with LAMs (*Gpnmb* ^pos^, *Mmp12* ^neg^), suggesting that FPR2 could be involved in *Mmp12* upregulation in monocyte-derived macrophages.

We next tested the role of FPR2 in SAA-induced pro-resolving changes in macrophages (Figure 8*D*). We found that knockdown of *Fpr2* in macrophages significantly reduced *Mmp9, Mmp12, Ctsd,* and *Psap* gene expression in the presence of recombinant SAA (Figure 8*D*). In addition, *Fpr2* knockdown promoted *Tnf* gene expression, which was exacerbated in the presence of SAA; and promoted *Tgfb1* gene expression when SAA was present (Figure 8*D*), suggesting that SAA might have pro-inflammatory roles via other receptors, when FPR2 is not present.

Taken together, hepatocyte-produced SAA promotes pro-resolving macrophage phenotype changes in FPR2-dependent way.

### Recombinant SAA Supplementation Promotes Fibrosis Resolution After Alcohol Cessation

**Figure 9:**
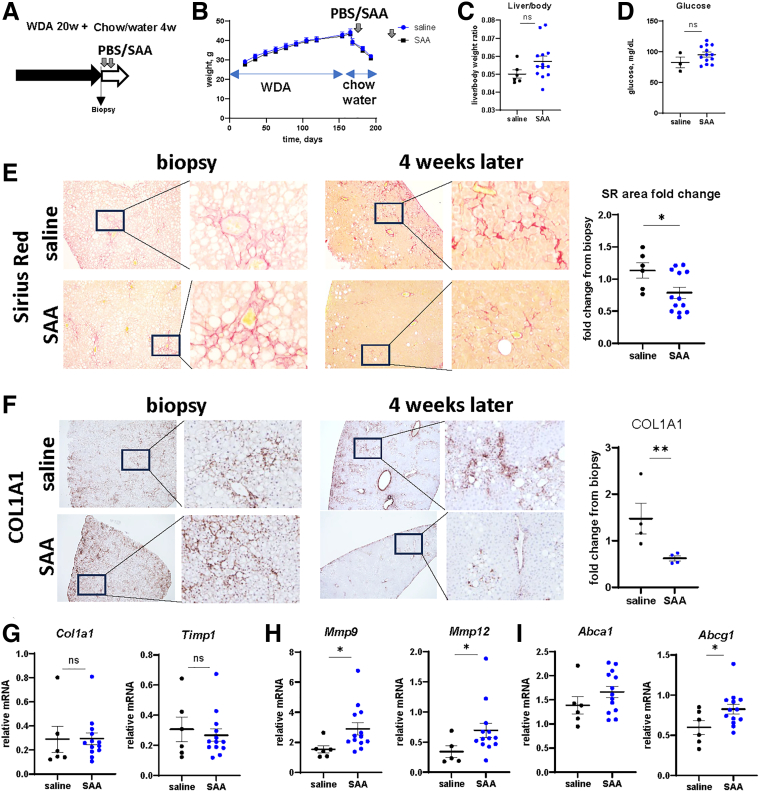
**SAA supplementation promotes fibrosis resolution in vivo.** (*A*) Mice were fed WDA for 20 weeks. Liver biopsy was collected, and mice were placed on chow diet with plain water for 4 weeks. Mice were treated with recombinant SAA fragments (3 μg/mouse) twice after alcohol cessation. (*B*) Weight change in these mice. (*C*) Liver to body weight ratio at the end of the experiment. (*D*) Fasting blood glucose levels at the end of the experiment. (*E*) Skin rash scores (0 – none, 1 – mild, 2 – severe). (*F*) Representative images of Sirius Red staining and COL1A1 staining in control and treated mice showing biopsy and liver after 4 weeks from the same mouse. *Right.* Fold change in positive area from biopsy sample. N = 6–13 mice per group. ∗*P* < .05. (*G–I*) Relative gene expression changes (fold change from biopsy sample) in these mice. N = 6–13 mice per group. ∗*P* < .05.

To assess the ability of SAA to promote fibrosis resolution in vivo, we supplemented mice with recombinant SAA. Mice were fed WDA for 20 weeks, and a small liver biopsy was collected at the end of the feeding to control for disease severity. Mice were subsequently placed on chow diet with plain water for 4 weeks and received intraperitoneal injections of recombinant SAA (3 μg/mouse) 1 week and 2 weeks after alcohol cessation (Figure 9*A*). SAA did not affect weight changes in these mice (Figure 9*B*), liver to body weight ratio (Figure 9*C*), or fasting glucose at the end of the experiment (Figure 9*D*). However, a subset of mice (3/13) developed moderate to severe skin rashes after SAA treatment, suggesting that SAA may have unwanted systemic effects.

We found that SAA treatment promoted fibrosis resolution evident by a decrease of Sirius Red and COL1A1 staining after 4 weeks of resolution (Figure 9*E–F*). Fibrogenic genes (*Col1a1, Timp1*) were downregulated 4 weeks after alcohol cessation to the same levels in control and SAA-treated mice (Figure 9*G*). In contrast, MMP gene expression was significantly induced in SAA-treated mice (Figure 9*H*). Previously, we reported that liver X receptor (LXR)α activation is an important mechanism upstream of MMP activation required for fibrosis resolution in ALD. We found that SAA treatment induced LXRα target gene expression (Figure 9*I*), suggesting that MMP induction could be mediated by LXRα activation in liver macrophages. Taken together, these data show that SAA supplementation promotes fibrosis resolution in vivo.

### SAA Associates With HDL During Fibrosis Resolution

**Figure 10:**
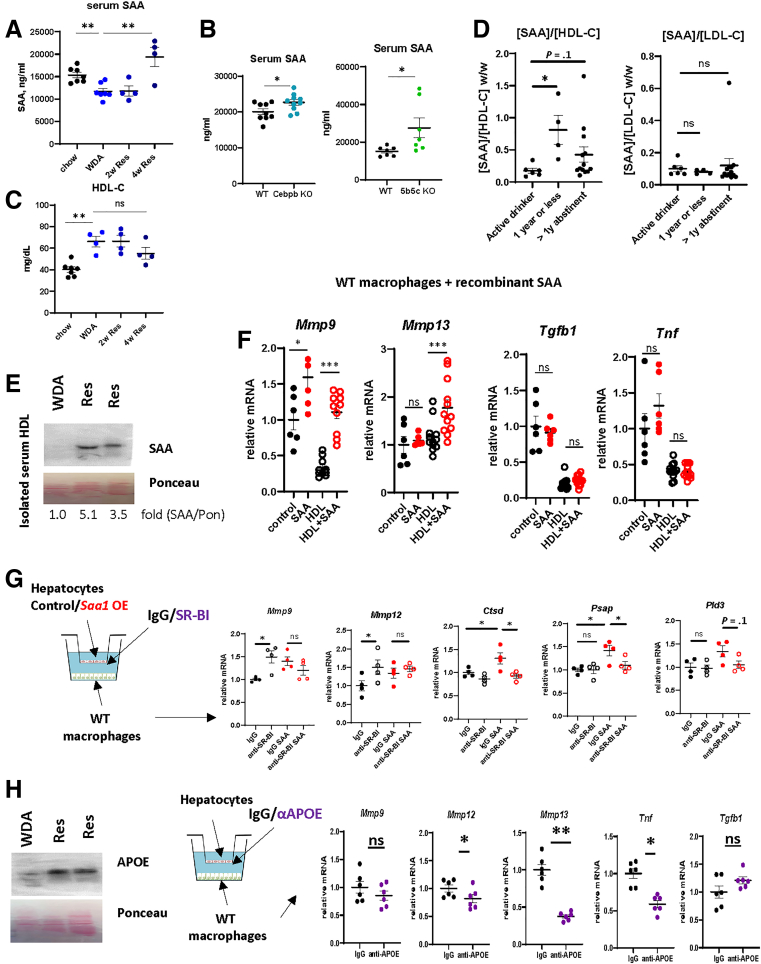
**SAA pro-resolving activity requires HDL.** (*A*) SAA serum levels in chow-fed mice, or mice fed WDA at the end of the feeding or at 2 or 4 weeks after alcohol cessation. N = 4–7 mice per group. ∗∗*P* < .01. (*B*) Serum SAA in WT mice or *Cebpb* KO and *Kdm5b/Kdm5c* KO mice after 4 weeks of resolution. N = 7–10 mice per group. ∗*P* < .05. (*C*) HDL-C serum levels in chow-fed mice, or mice fed WDA at the end of the feeding or at 2 or 4 weeks after alcohol cessation. N = 4–7 mice per group. ∗∗*P* < .01. (*D*) Serum SAA to HDL-C or to LDL-C ratios in patients with ALD cirrhosis either active drinkers (N = 6), alcohol abstinent for 1 month to 1 year (N = 4), or more than a year abstinent (N = 13). ∗*P* < .05. (*E*) HDL was isolated from serum of the mice fed WDA or mice at 4 weeks after alcohol cessation. Western blot analysis of purified HDL. (*F*) HDL was purified from hepatocytes conditioned media. Liver macrophages were treated with recombinant SAA (1 μg/mL) in serum-free media in the presence or absence of HDL (25 μg/mL). N = 6–10. ∗*P* < .05; ∗∗∗*P* < .001. (*G*) Hepatocytes were transfected with control vector or vector expressing mouse *Saa1* and were used in a co-culture system with liver macrophages from chow-fed mice. Hepatocyte-macrophage co-cultures were supplemented with neutralizing antibodies specific for SR-BI or IgG control. N = 4. ∗*P* < .05. (*H*) *Left*. Western blot analysis of purified HDL. *Right.* Hepatocyte-macrophage co-cultures were supplemented with neutralizing antibodies specific for APOE or IgG control. N = 6. ∗*P* < .05; ∗∗*P* < .01; ∗∗∗*P* < .001.

In circulation, SAA associates with HDL displacing APOA1 and altering HDL function, cargo, and fate.^9^^,^^10^^,^^13^ To assess the functional consequences of SAA association with HDL, we examined serum SAA-HDL levels during ALD resolution. First, we assessed total serum SAA levels using an enzyme-linked immunosorbent assay (ELISA) (Figure 10*A* and *B*). In agreement with hepatocyte gene expression changes, circulating SAA levels were induced about 2-fold 4 weeks after alcohol cessation (Figure 10*A*). Serum SAA was further elevated in hepatocyte-specific *Cebpb* KO and *Kdm5b* KO mice at 4 weeks of resolution (Figure 10*B*). In contrast, HDL-C levels were not significantly altered during disease resolution (Figure 10*D*) resulting in elevated SAA/HDL-C.

We examined serum samples from patients with ALD cirrhosis. We found that total serum SAA were not significantly altered with abstinence; however, SAA/HDL-C ratios (but not SAA/low-density lipoprotein cholesterol [LDL-C]) were increased in abstinent patients especially in <1 year group, suggesting that SAA may be enriched on HDL particles after abstinence (Figure 10*D*).

We next isolated serum HDL from WDA and 4-week resolution mice and analyzed SAA protein abundance by Western blot (Figure 10*E*). We found that SAA is enriched on HDL particles after alcohol cessation. Next, we tested whether HDL is required for SAA function. Isolated mouse macrophages were treated with recombinant SAA alone, in serum-free media, or in the presence of HDL (Figure 10*F*). We found that SAA alone promoted *Mmp9* gene expression 1.5-fold but did not affect other genes. In contrast, in the presence of HDL, SAA treatment greatly induced *Mmp9* and *Mmp13* gene expression (Figure 10*F*), suggesting that HDL modulates the action of SAA.

In agreement with these data, blocking HDL uptake by SR-BI neutralizing antibody treatment prevented SAA-mediated pro-resolving changes in liver macrophages (Figure 10*G*). In the presence of IgG control, *Saa1* expression in hepatocytes stimulated *Mmp9*, *Mmp12, Ctsd, Psap,* and *Pld3* in macrophages; in contrast, in the presence of SR-BI antibody, *Saa1* expression in hepatocytes failed to induce these genes. Interestingly, we found that in the absence of SAA, HDL uptake suppresses MMP gene expression, suggesting that SAA stimulates MMPs in part by preventing HDL-mediated MMP inhibition.

Apolipoprotein E (APOE), a marker of acute phase HDL, was similarly enriched on resolution HDL compared with HDL from alcohol-fed mice. Similarly to SAA depletion, APOE depletion in a hepatocyte-macrophage co-culture system reduced macrophage *Mmp12* and *Mmp13* gene expression (Figure 10*H*), suggesting that acute phase HDL promotes the pro-resolving macrophage phenotype.

Taken together, SAA’s ability to induce pro-resolving macrophage changes depends on HDL and SR-BI-mediated HDL uptake by macrophages.

### SAA-enriched HDL Supplementation Stimulates Fibrosis Resolution In Vivo Via Niche Remodeling

**Figure 11:**
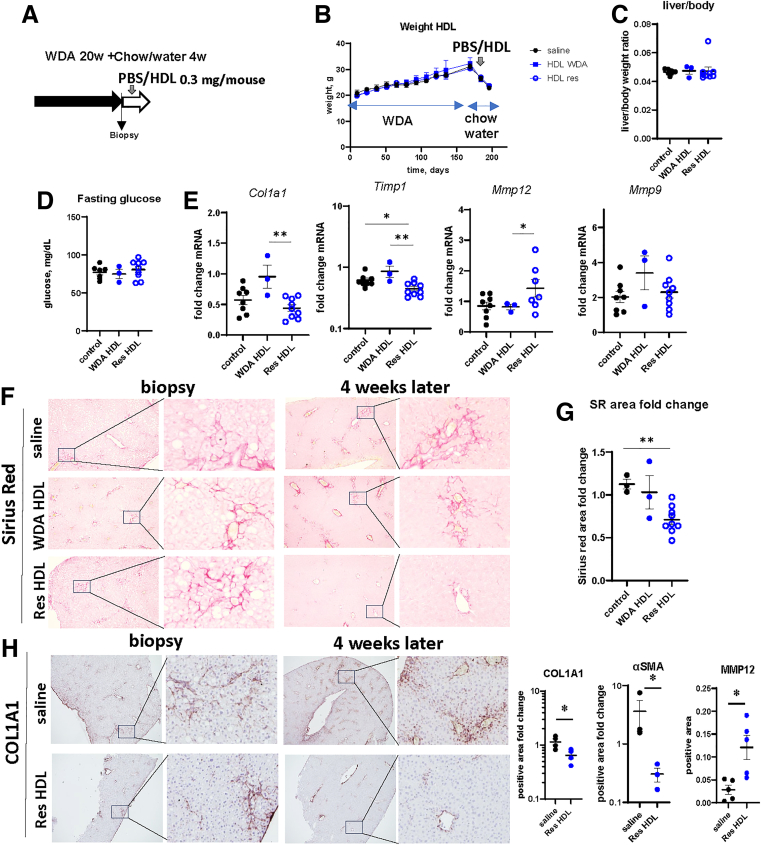
**SAA-enriched HDL supplementation promotes fibrosis resolution in vivo.** (*A*) HDL was isolated from serum of the mice fed WDA (WDA HDL) or mice at 4 weeks after alcohol cessation (Res HDL). Mice were fed WDA for 20 weeks. Liver biopsy was collected, and mice were placed on chow diet with plain water for 4 weeks. Mice were treated with HDL (0.3 mg/mouse) or saline control 1 week after alcohol cessation. (*B*) Weight change in these mice. (*C*) Liver to body weight ratio at the end of the experiment. (*D*) Fasting blood glucose levels at the end of the experiment. (*E*) Relative gene expression changes (fold change from biopsy sample) in these mice. N = 3–9 mice per group. ∗*P* < .05; ∗∗*P* < .01. (*F*) Representative images of Sirius Red staining in control and treated mice showing biopsy and liver after 4 weeks from the same mouse. (*G*) Fold change in positive area from biopsy sample. N = 3–9 mice per group. ∗∗*P* < .01. (*H*) Representative images of COL1A1 staining in control and treated mice showing biopsy and liver after 4 weeks from the same mouse. *Right.* Fold change in positive area from biopsy sample. N = 3–4 mice per group. ∗*P* < .05.

**Figure 12:**
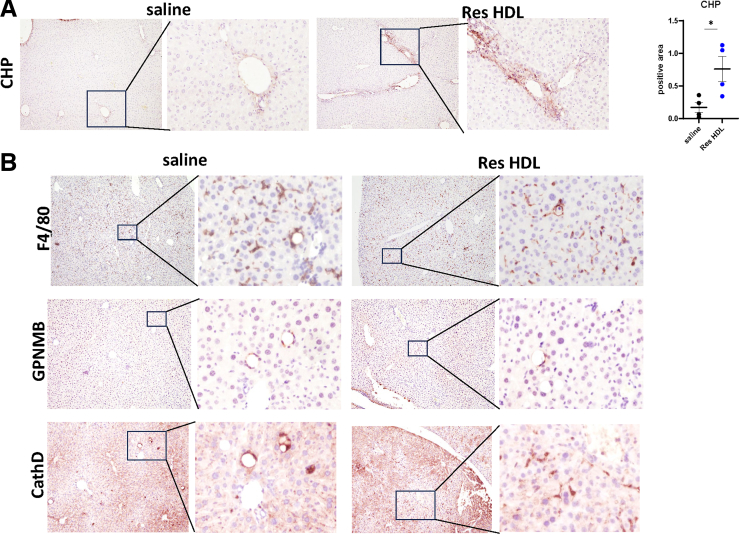
**SAA-enriched HDL supplementation promotes macrophage collagen remodeling ability.** Mice were fed WDA for 20 weeks. Liver biopsy was collected, and mice were placed on chow diet with plain water for 4 weeks. Mice were treated with HDL (0.3 mg/mouse) or saline control 1 week after alcohol cessation. (*A*) CHP staining in in control and treated mice at 4 weeks after alcohol cessation. *Right.* CHP-positive area. N = 4. ∗*P* < .05. (*B*) Representative images of immunohistochemistry staining in control and treated mice showing biopsy and liver after 4 weeks from the same mouse.

SAA upregulation in hepatocytes and on HDL particles occurs only after 4 weeks of alcohol cessation (Figure 10*A*). We hypothesized that earlier supplementation with SAA-HDL might stimulate resolution. To test that, we treated mice 1 week after alcohol cessation with isolated HDL from WDA-fed mice (WDA HDL) or from mice 4 weeks after alcohol cessation (Res HDL) (0.3 mg/mouse) or phosphate buffered saline (PBS) control (Figure 11*A*). HDL treatment did not affect weight changes (Figure 11*B*), liver to body weight ratio (Figure 11*C*), or fasting glucose (Figure 11*D*) at the end of the feeding. However, Res HDL did promote pro-resolving gene expression changes in the liver compared with WDA HDL (Figure 11*E*). Res HDL treatment promoted a significant reduction in liver fibrosis assessed by Sirius Red staining (Figure 11*F* and *G*) and COL1A1, αSMA staining (Figure 11*H*). At the end of the experiment, mice showed increased collagen remodeling assessed by CHP labeling (Figure 12*A*). In addition, we observed altered distribution of cathepsin D, marker of pro-resolving macrophages identified before. In control animals, cathepsin D marked crown-like structures, whereas in Res HDL-treated animals, cathepsin D-positive cells were more numerous and distributed throughout liver parenchyma (Figure 12*B*), suggesting that Res HDL promoted a pro-resolving shift in liver macrophages, thus enhancing fibrosis resolution.

**Figure 13:**
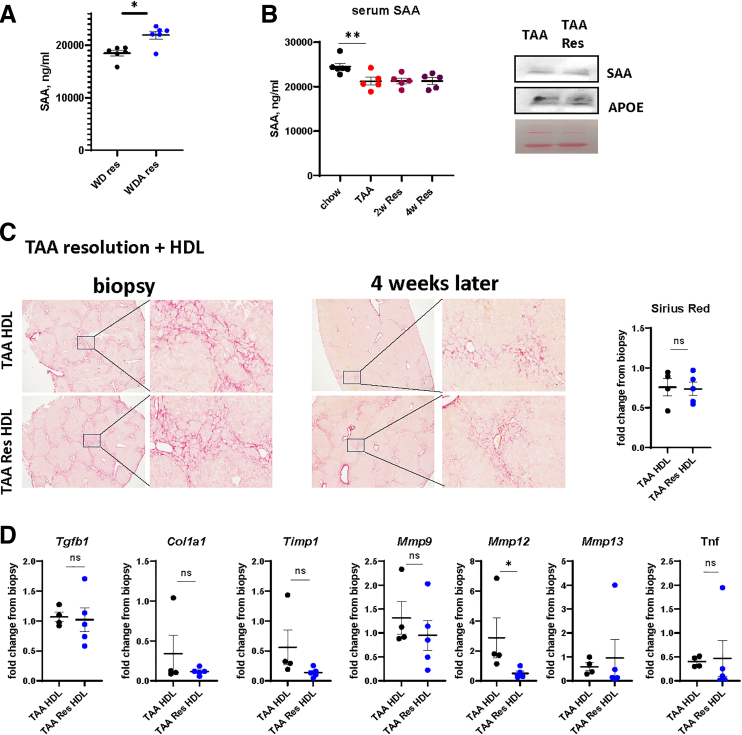
**Pro-resolving HDL remodeling does not occur in TAA fibrosis resolution.** (*A*) Serum SAA levels in mice fed WD for 30 weeks followed by 4 weeks of resolution (WD res) and in mice treated with WDA for 20 weeks followed by 4 weeks resolution (WDA res). N = 6 mice per group. ∗*P* < .05. (*B*) *Left.* Serum SAA levels in mice treated with TAA or at 2 and 4 weeks after TAA removal. *Right.* HDL was isolated from serum of the mice fed TAA (TAA HDL) or mice at 4 weeks after TAA cessation (TAA Res HDL). Western blot analysis of purified HDL. (*C*) Mice were treated with TAA for 10 weeks. Liver biopsy was collected, and mice were given plain water for 4 weeks. Mice were treated with HDL (0.3 mg/mouse) 1 week after alcohol cessation. Representative images of Sirius Red staining in mice showing biopsy and liver after 4 weeks from the same mouse. *Right.* Fold change in positive area from biopsy sample. N = 4–5 mice per group. (*D*) Relative gene expression changes (fold change from biopsy sample) in these mice. N = 4–5 mice per group. ∗*P* < .05.

We next tested whether SAA-mediated resolution occurs in other models of liver fibrosis. To evaluate the role of SAA in metabolic dysfunction-associated steatohepatitis (MASH) resolution, we compared serum samples from WD (30 weeks) and WDA (20 weeks) followed by 4 weeks resolution, from a previously reported experiment.^20^ We found that WDA resolution had significantly higher SAA compared with WD resolution, suggesting that WD withdrawal alone does not induce SAA upregulation (Figure 13*A*). In addition, we found that TAA resolution did not increase SAA levels and SAA-HDL levels (Figure 13*B*), and HDL from TAA resolution mice did not affect fibrosis resolution compared with control HDL from TAA-treated mice (Figure 13*C* and *D*). Taken together, these data indicate that ALD resolution specifically induces HDL remodeling that promotes a pro-resolving HDL shift.

### Interleukin-22 Promotes SAA Induction in the Liver After Alcohol Cessation

**Figure 14:**
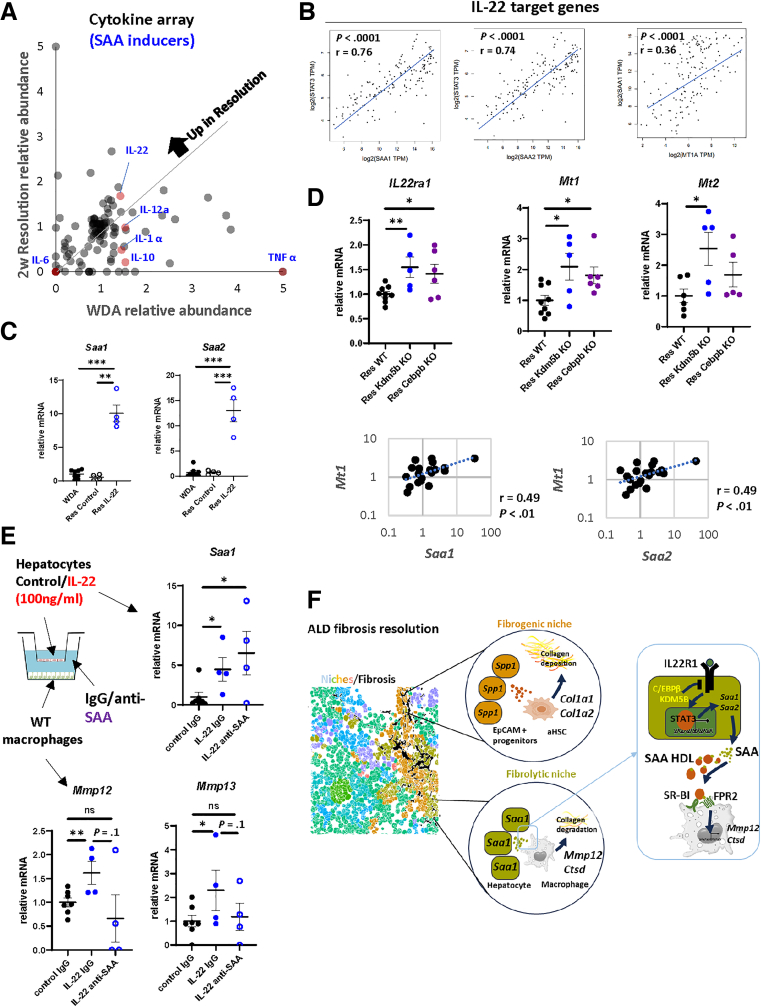
**IL-22 promotes SAA induction after alcohol cessation.** (*A*) Serum cytokine array in mice fed WDA or at 2 weeks after alcohol cessation. (*B*) Correlation between *SAA1, SAA2* and *STAT3,* or *MT1A* gene expression in human liver samples (TCGA control and GTEx). (*C*) Relative liver mRNA in WDA-fed mice and in mice 4 weeks after alcohol cessation. Recombinant mouse IL-22 was administered at 200 μg/kg every 4 days for 4 weeks after WDA cessation. N = 4. ∗∗∗*P* < .001. (*D*) Relative gene expression in WT, *Kdm5b* KO, or *Cebpb* KO mice at 4 weeks after alcohol cessation. N = 6–9. ∗*P* < .05; ∗∗*P* < .01. *Bottom.* Correlation between *Saa1/2* and *Mt1* gene expression in these mice. (*E*) Hepatocytes and liver macrophages were isolated from chow-fed mice. Hepatocyte-macrophage co-cultures were supplemented with 100 n/mL of IL-22 in the inserts, and neutralizing antibodies specific for SAA or IgG control (0.5 μg/mL) as indicated. N = 4–7. ∗*P* < .05; ∗∗*P* < .01. (*F*) Model of APR mediated niche remodeling after alcohol cessation.

SAA can be induced by a variety of cytokines including tumor necrosis factor (TNF)α, interleukin (IL)-1β, IL-6, and IL-22. We assessed changes in these upstream mediators after alcohol cessation using previously reported circulating cytokine array data from mice fed WDA and at 2 weeks after alcohol cessation (2w Resolution).^20^ We found that all detected pro-inflammatory cytokines were reduced after alcohol cessation, whereas IL-22 was slightly elevated (Figure 14*A*). In the liver, IL-22 upregulates genes that promote survival, proliferation, and regeneration.^27^ IL-22 stimulates hepatocyte expression of MT-1 and MT-2 to protect from oxidative stress. We found that *MT1A* and the IL-22-associated transcription factor *STAT3* both strongly correlated with *SAA1* and *SAA2* expression in human liver samples, suggesting that SAA genes could be direct targets of IL-22/STAT3 signaling in the liver (Figure 14*B*). Indeed, when mice were treated with recombinant mouse IL-22 at 200 μg/kg every 4 days for 4 weeks after alcohol cessation, we observed an 18-fold induction for both *Saa1* and *Saa2* compared with untreated control (Figure 14*C*). Because *Kdm5b* and *Cebpb* KO mice had elevated expression of SAA, we examined IL-22 pathway in these mice. We found that both hepatocyte-specific *Kdm5b* and *Cebpb* KO mice had elevated *Il22ra1* expression and IL-22 target gene expression (*Mt1, Mt2*), suggesting that IL-22 signaling induction could contribute to SAA upregulation in these mice. In agreement with this hypothesis, we found that *Mt1* expression strongly correlated with *Saa1* and *Saa2* gene expression in livers of these mice (Figure 14*D*). Taken together, these data suggest that KDM5B and C/EBPβ-mediated IL-22 receptor downregulation suppressed IL-22 mediated signaling, and SAA induction, thus preventing SAA-mediated pro-resolving changes.

To confirm that IL-22 signaling can promote pro-resolving changes in liver macrophages via SAA upregulation, we treated hepatocyte-macrophage co-cultures with recombinant IL-22 in the presence or absence of SAA neutralizing antibody (Figure 14*E*). We found that IL-22 induced a 5-fold upregulation of *Saa1* in hepatocytes and *Mmp12* and *Mmp13* in macrophages. SAA antibody added to the co-culture completely abolished MMP upregulation, suggesting that SAA mediated the IL-22 effect on MMPs.

Taken together, the results show that IL-22 dependent signaling controls SAA upregulation after alcohol cessation in the liver; moreover, IL-22 mediated induction of SAA in hepatocytes promotes pro-resolving changes in liver macrophages.

## Discussion

ALD is the main cause of alcohol-related mortality.^28^ Despite decades of studying molecular mechanisms involved in disease progression, therapeutic approaches are still limited.^28^ Alcohol cessation is the most beneficial intervention for patients with ALD,^1^^,^^2^^,^^29^ but recovery can be slow for those with severe disease, and some patients have progressive cirrhosis even after abstinence.^1^^,^^2^^,^^29^

Liver function is regulated by extensive interactions between hepatocytes and NPCs. These cells exchange signals in their local milieu and thus establish discrete, functionally distinct, cellular niches within the liver responsible for processes such as fibrogenesis or fibrolysis (Figure 14*F*). Our spatial transcriptomics analysis in mice after alcohol cessation defines cellular components of these niches and potential mediators of cell-to-cell communication within these niches.

One of the findings from this work is that *Epcam*^pos^ progenitors are an important component of the fibrogenic niche in alcohol-fed mouse livers. Immature progenitors derived from hepatocytes (ductular reaction) are dramatically increased in ALD livers and promote disease progression as well as prevent disease resolution.^30^ They can signal to activated stellate cells within the fibrogenic niche to promote collagen production via mediators such as osteopontin,^31^, ^32^, ^33^ whereas stellate cell-mediated signals may maintain the progenitor immature state. Interestingly, although the fibrogenic niche was dramatically reduced after alcohol cessation, another niche (niche 7) containing *Epcam*^pos^ progenitors and *Fabp4*^pos^ endothelial cells (cluster 21) persisted after alcohol cessation. The fate and function of ductular reaction after alcohol cessation needs further investigation.

The main finding of this work is that hepatocyte APR signaling is a central hub of the fibrolytic niche in the liver (Figure 14*F*). APR-expressing hepatocytes co-localize with pro-resolving liver macrophages in mice and humans and can induce pro-resolving changes in naïve macrophages in vitro. Furthermore, we identified SAA as one of the key contributors to APR-mediated effects. SAA is necessary and sufficient to induce pro-resolving changes in macrophages as well as the ECM remodeling required for fibrosis resolution in vitro and in vivo.

Other acute phase proteins such as PLG, LBP, SERINA3, and FGL-1 have also been shown to contribute to fibrosis resolution. PLG can bind MARCO and Plg-RKT to enhance macrophage phagocytosis.^34^ LBP acts commonly through TLR4 and CD14, and LBP can also interact with macrophages independently of LPS and CD14, potentially influencing intracellular LPS signaling,^35^ SERPINA3 can modulate multiple inflammatory pathways and WNT signaling,^36^ and FGL-1 promotes liver regeneration and modulates immune pathways in the liver. Determining the relative contributions of individual APPs in fibrosis resolution will require further investigation, but based on our findings, SAA has one of the strongest effects.

Once secreted, SAA rapidly associates with HDL and other lipoprotein particles.^9^^,^^37^ SAA-HDL has altered cargo (both lipid and protein), altered half-life in circulation, and altered cellular uptake.^8^^,^^9^^,^^18^^,^^37^^,^^38^ Some studies suggest that SAA-HDL is more likely to be taken up by macrophages.^17^^,^^38^ Our data suggest that SAA-HDL can play a role in hepatocyte-macrophage crosstalk involved in ALD resolution. Compared with control HDL, SAA-HDL stimulated MMP gene expression in macrophages as well as the expression of genes such as cathepsin D, a marker of pro-resolving macrophages required for fibrosis resolution.^23^ Hepatocyte-produced HDL was previously demonstrated to modulate fibrosis of the liver, lung, and kidney.^39^^,^^40^ Clinical studies in metabolic liver disease (MASH) resolution have shown that increased HDL correlates with disease resolution^41^ and that the content of HDL controls its various protective functions such as tissue repair and fibrosis resolution.^39^ We found that SAA-HDL had potent pro-resolving properties in vivo, promoting fibrosis resolution by inducing MMP expression and macrophage phenotype shifts. Taken together, these findings show that acute-phase HDL, enriched in SAA, promotes fibrosis resolution after alcohol cessation. This may have important clinical implications for patients with ALD.

It is not clear whether this mechanism is specific to ALD. We found that in the TAA-induced fibrosis model, HDL remodeling is not involved in fibrosis resolution, suggesting that this mechanism may not be relevant in all forms of liver fibrosis, particularly those that are not associated with liver steatosis. Whether this mechanism is present in metabolic dysfunction-associated steatotic liver disease (MASLD) resolution is not clear.

The mechanism of SAA-mediated macrophage phenotype changes has been previously investigated and is likely context-dependent. Previous studies showed that SAA can bind the FPR2 receptor,^15^^,^^16^ TLR2,^12^ RAGE, and CD36.^42^ SAA-HDL can be taken up via interaction with SR-BI. FPR2, also known as a resolvin D1 receptor, is required for some of SAA’s downstream effects in myeloid cells, such as MMP induction.^42^ We found that macrophage FPR2 mediated SAA-dependent pro-resolving changes in liver macrophages. Thus, FPR2 activation could be another potential target for fibrosis resolution in ALD.

The mechanism of APR activation in response to alcohol cessation is not fully defined. We found that APR activation after alcohol cessation occurred in WT mice but was greatly enhanced in *Kdm5b* and *Cebpb* KO mice, 2 mouse models of increased fibrosis resolution we described previously. C/EBPβ is known to regulate APR gene expression directly^10^, ^11^, ^12^^,^^14^; however, other transcription factors such as STAT3 and NF-κB can induce APR genes downstream of cytokines such as IL-1β, IL-6, and IL-22.^43^^,^^44^ Our data suggest that C/EBPβ-KDM5B action antagonizes APR induction after alcohol cessation via suppression of *Il22ra1*. Both *Kdm5b* and *Cebpb* KO as well as IL-22 supplementation greatly induced IL-22 signaling and SAA upregulation in the liver. IL-22 is an emerging target for liver disease treatment that can promote hepatocyte regeneration. Our studies suggest that IL-22 mediated APR activation may be involved in fibrosis resolution as well.

Taken together, we have uncovered a complex cell-cell communication network during ALD development and resolution that involves APR activation in hepatocyte. Targeting APR upstream (KDM5B, C/EBPβ, IL22) and downstream (HDL, FPR2) signaling events could be a promising novel strategy to accelerate or enhance ALD resolution after alcohol cessation.

## Materials and Methods

### Mice

*Kdm5b* floxed mice (B6/J^GptKdm5bem1C^ flox/wt) were obtained from GemPharmatech Co., Ltd and bred to make homozygous flox/flox breeders. *Cebpb* floxed mice (BALB/cJ-Cebpb^tm1.1Elgaz^) were obtained from Jackson Laboratory and backcrossed for 7 generations to the C57BL6/J background.

All mice were housed in a temperature-controlled, specific pathogen-free environment with 12-hour light-dark cycles. All animal handling procedures were approved by the Institutional Animal Care and Use Committee at the University of Kansas Medical Center.

To induce hepatocyte-specific KO, mice were treated with adeno-associated virus (AAV)-TBG-control and AAV-TBG-iCre (VectorBiolabs, Malvern, PA) at 1 × 10^11^ genome copies per mouse at the time of alcohol cessation.

### WDA-resolution Model

Both male and female mice were fed ad libitum Western diet (Research Diets, Inc, Cat# D12079B), and alcohol was given ad libitum in water as described previously.^21^ Mice received progressively increasing amount of alcohol in water (1%, 3%, 10%, 15%, and 20% for 3 days each). After reaching 20%, mice continued for 16 to 20 weeks. At the end of the feeding, a small liver biopsy was collected, and mice were placed on chow diet with plain water for 4 weeks.

### Biopsy

Small liver biopsies were collected as previously described.^45^ Mice were anesthetized with isoflurane; a small incision was made on the upper right side of the abdomen and the liver exposed. One lobe of the liver was carefully lifted, and a small piece of liver (3–5 mm) was excised with scissors. The tissue was immediately placed in zinc-formalin and RNA-later for further processing. The gap in the liver was closed with an absorbable hemostatic gelatin sponge (Vetspon #96002). The incision was closed with 5-0 absorbable surgical suture (Redilene Redisorb Fast Pro #VF493-M) and 7-mm wound clips (Reflex 7 #203-1000). Mice were then injected with 1 mL saline (subcutaneously [SC]) and 1.0 mg/kg SR buprenorphine (SC). Mice were placed on a heating pad and monitored until fully awake from anesthesia; thereafter, mice were monitored daily for the next 7 days. The wound clips were removed on day 8 after surgery.

### Vectors

FPR2 small interfering RNA (siRNA) (m) (cat# sc-145234), SAA siRNA (cat# sc-40818), and control siRNA (cat# sc-37007) were from SantaCruz.

### Cell Isolation

Liver cells were isolated by a modification of the method described by Troutman et al.^46^ Mouse livers were digested by retrograde perfusion with liberase via the inferior vena cava. The dissociated cell mixture was placed into a 50-mL conical tube and centrifuged twice at 50 *g* for 2 minutes to pellet hepatocytes. The NPC-containing cell supernatant was further used to isolate macrophages. The cell suspension was pelleted by centrifugation (700 g, 10 minutes, 4°C) and resuspended in PBS and OptiPrep (Sigma) to a final concentration of 17%. Afterwards, 5 mL of the indicated suspension was placed in a 15-mL polystyrene conical centrifuge tube (BD Biosciences) and overlaid with 5 mL of a 9% Optiprep solution followed by 2 mL PBS. After centrifugation at 1400 g for 20 minutes at 4°C with decreased acceleration and without breaks, the various cell types were arranged according to their density. HSCs were enriched in the upper cell layer, whereas KCs and LSECs were separated as a second layer of higher density. Cell fractions were collected separately by pipetting. The KC/LSEC fraction was pelleted, and macrophages were isolated with F4/80+MicroBeads (MiltenyiBiotec) according to the manufacturer’s instructions. Cells were applied onto LS magnetic-activated cell sorting (MACS) columns (MiltenyiBiotec), which were placed within the magnetic field of a MACS separator and washed 3 times with MACS buffer (MiltenyiBiotec). Cells were eluted and then seeded into culture dishes.

### Collagen Degradation Assay

Collagen degradation assay was performed using DQ Collagen (ThermoFisher, Cat# D12060), type I From Bovine Skin, Fluorescein Conjugate, according to the manufacturer’s instructions. Cells were incubated in 50 mM Tris-HCl (pH 7.6), 150 mM NaCl, 5 mM CaCl_2_, and 10 mg/L DQ Collagen for 2 hours at 37°C. Digestion product fluorescence emission was measured at 525 nm.

### Immunohistochemistry

Liver tissue sections (5-μm thick) were prepared from formalin-fixed, paraffin-embedded (FFPE) samples. Immunostaining on formalin-fixed sections was performed by deparaffinization and rehydration followed by antigen retrieval by heating in a pressure cooker (121°C) for 5 minutes in 10 mM sodium citrate, pH 6.0 as described previously.^47^ Peroxidase activity was blocked by incubation in 3% hydrogen peroxide for 10 minutes. Sections were rinsed 3 times in PBS/PBS-T (0.1% Tween-20) and incubated in Dako Protein Block (Dako) at room temperature for 1 hour. After removal of blocking solution, slides were placed into a humidified chamber and incubated overnight with a primary antibody, diluted 1:300 in Dako Protein Block at 4°C. Antigen was detected using the SignalStain Boost IHC detection reagent (catalogue # 8114; Cell Signaling Technology, Beverly, MA), developed with diaminobenzidene (DAB; Dako), counterstained with hematoxylin (Sigma-Aldrich), and mounted.

### Western Blotting

Protein extracts (50 μg) were subjected to 10% sodium dodecyl sulfate-polyacrylamide gel electrophoresis (SDS-PAGE), electrophoretically transferred to nitrocellulose membranes (Amersham Hybond ECL, GE Healthcare), and blocked in 3% bovine serum albumin (BSA)/PBS at RT for 1 hour. Primary antibodies were incubated overnight at manufacturer-recommended concentrations. Immunoblots were detected with the ECL Plus Western Blotting Detection System (Amersham Biosciences) or using near-infrared fluorescence with the ODYSSEY Fc, Dual-Mode Imaging system (Li-COR).

### RT-PCR

RNA was extracted from livers using the RNeasy Mini Kit (Qiagen). cDNA was generated using the RNA reverse transcription kit (Applied Biosystems, Cat.No 4368814). Quantitative real time RT-PCR was performed in a CFX96 Real time system (Bio-Rad) using specific sense and antisense primers (Table 1) combined with iQ SYBR Green Supermix (Bio-Rad) for 40 amplification cycles: 5 seconds at 95°C, 10 seconds at 57°C, and 30 seconds at 72°C. mRNA concentrations were calculated relative to *Actb*.

### Primers

**Table:** Table 1. Primers

mActb fwd	ATGTCACGCACGATTTCCCT
mActb rvs	CGGGACCTGACAGACTACCT
mTnf fwd	CTGAGACATAGGCACCGCC
mTnf rvs	CAGAAAGCATGATCCGCGAC
mCol1a1 fwd	TGGCCAAGAAGACATCCCTG
mCol1a1 rvs	GGGTTTCCACGTCTCACCAT
mMmp9 fwd	CCCTGGAACTCACACGACAT
mMmp9 rvs	TCACACGCCAGAAGAATTTGC
mTimp1 fwd	GTAAGGCCTGTAGCTGTGCC
mTimp1 rvs	AGCCCTTATGACCAGGTCCG
mAbca1 fwd	AGGACTAGACTCCAAGTTCTTCA
mAbca1 rvs	TGGACACCTTCTATGACAATTCTAC
mAbcg1 fwd	TGTCAGATACGGCTTTGAGGG
mAbcg1 rvs	GATGTCGCAGTGCAGGTCTT
mTgfb1 fwd	TACGTCAGACATTCGGGAAGC
mTgfb1 rvs	TTTAATCTCTGCAAGCGCAGC
mCcl2 fwd	ACCTGGATCGGAACCAAATGAG
mCcl2 rvs	GCTGAAGACCTTAGGGCAGAT
mCd163 fwd	GCTGAGGATGTCGGTGTGAT
mCd163 rvs	TCCTGAACATCTGGACACTCC
mMmp12 fwd	GTGGTACACTAGCCCATGCTT
mMmp12 rvs	TCCACGTTTCTGCCTCATCAA
mMmp13 fwd	ATGAAGACCCCAACCCTAAGC
mMmp13 rvs	ATGGCATCAAGGGATAGGGC
mLcn2 fwd	TGGCGAACTGGTTGTAGTCC
mLcn2 rvs	CACTCTGGGAAATATGCACAGG
mSerpine1 fwd	TTGTGCCGAACCACAAAGAG
mSerpine1 rvs	AGGCACTGCAAAAGGTCAGG
mFgl1 fwd	TGGCCTAATTTTCATAACCACAGA
mFgl1 rvs	AGGGCAGAAACGGATAATGGT
mPlg fwd	AGTCCTCAGCATCACCAGAC
mPlg rvs	TGGTAGCATTCCTGGACCAC
mLbp fwd	ACAAGATCACACTACCGGACT
mLbp rvs	GAAACTCGTACTGCCCACGA
mSerpina3n fwd	TTAGTTCCCAGCTGACCAACC
mSerpina3n rvs	TTTGCTCCTACCTCTTTATTCTGGT
mC9 fwd	AGGGAGCAAGCAATTCTCCT
mC9 rvs	CCAGTTGGCGAAGTCAGTCT
mCrp fwd	GTGGGTGGTGCTGAAGTACG
mCrp rvs	AATCCCCGTAGCAGACTCCC
mCtsd fwd	CTCCCCGTGGTAGTACTTGG
mCtsd rvs	TACCTGAACAGGGACCCAGAA
mPsap fwd	CCTGGACATGATTAAGGGCGA
mPsap rvs	TACTCCTGAAGGGACTGGCA
mPld3 fwd	TTGTGGTTGACACGGGCATA
mPld3 rvs	CTCACTCCGACATCCAGGTG
mAbca1 fwd	AGGACTAGACTCCAAGTTCTTCA
mAbca1 rvs	TGGACACCTTCTATGACAATTCTAC
mAbcg1 fwd	TGTCAGATACGGCTTTGAGGG
mAbcg1 rvs	GATGTCGCAGTGCAGGTCTT

### Transwell Co-culture

For co-culture experiments, primary mouse hepatocytes were placed in cell inserts of 24 well transwell plates (Corning Incorporated; 0.4 μm pore size) at a seeding density of 5 × 10^4^/well. Cells were treated as indicated. Freshly isolated liver macrophages were seeded in the bottom well at a seeding density of 1 × 10^4^/well. The cells were then cultured for 24 hours, and hepatocytes and macrophages were harvested for RNA isolation.

### Spatial Transcriptomics Analysis

Spatial transcriptomics was performed using CosMx Nanostring 1000-plex assay as a part of Technology Access Program. Assay was performed in CosMx Spatial Molecular Imager (SMI) using Mouse Universal Cell Characterization Panel, 1000-plex, RNA with Cell Segmentation Kit (DAPI Nuclear Stain, CD298/B2M, PanCK/CD45, CD68).

Five-micron tissue sections were cut from FFPE tissue blocks using a microtome, placed in a heated water bath, and adhered to Leica Bond Plus Microscope slides (Leica Biosystems). Slides were then dried at 37°C overnight and stored at 4°C.

Analysis was performed on 45 0.5 mm × 0.5 mm areas distributed evenly across 5 tissue sections. Areas (field of view [FOV]) were selected to include all liver zones, periportal, pericentral, midzonal areas, and areas of fibrosis and steatosis, as well as areas free of fibrosis. Sections were collected from mice fed WDA that produced high and low amount of fibrosis and matching resolution samples. No FOVs were excluded from analysis.

The SMI utilizes standard sample preparation methods typical for fluorescence in situ hybridization (FISH) or immunohistochemistry on FFPE tissue sections, followed by cyclic image acquisition and registration. Probe hybridization of ISH probes and antibodies and imaging on the SMI instrument for analyte readout and morphological imaging as well as initial bioinformatic analysis was performed by Nanostring (NanoString Technologies, Inc). Details of CosMx SMI chemistry, primary data processing. and filtering can be found in.^48^

Post-QC

No. FOVs: 45

Total tissue area (mm^2^): 9.89

Mean single-cell size (um^2^): 206.5

No. cells in final analysis: 47,899

Total transcripts: 40,737,436

Mean transcripts/cell: 850

Maximum transcripts/cell: 9927

Mean unique genes per cell: 269

Mean negative per cell: 0.016

Mean false codes per cell: 0.048

Single cell gene expression was then analyzed using Seurat R packages as previously described.^49^ Trajectory analysis was performed using Monocle3 as previously described.^3^

### CHP

CHP conjugates were from Advanced BioMatrix, CHP was labeled with biotin (B-CHP, Catalog No. 5265-60UG) for avidin/streptavidin-mediated detection, and was used at 20-μM concentration in 1% BSA according to manufacturer’s instructions.

Briefly, sections were deparaffinized, blocked with Fish Serum Blocking Buffer (ThermoFisher) followed by endogenous biotin blocking (biotin blocking kit, ThermoFisher). CHP was thermally dissociated at 80°C, quenched to room temperature, and added to tissue section overnight at 4°C. Tissue sections were then incubated with streptavidin-horseradish peroxidase (HRP) (5 μg/mL) for 30 minutes and developed with DAB (Dako), counterstained with hematoxylin (Sigma-Aldrich), and mounted.

### HDL Isolation

HDL was isolated using HDL Purification Kit (Cell Biolabs Inc) according to manufacturer’s instructions. HDL was dialyzed against PBS overnight and used at 20 μg/mL. HDL purity was confirmed by gel electrophoresis in denaturing and non-denaturing conditions.

### Human Specimens

De-identified human specimens were obtained from the Liver Center Tissue Bank at the University of Kansas Medical Center. Informed consent was obtained from all study participants. All studies using human tissue samples were approved by the Human Subjects Committee of the University of Kansas Medical Center.

### Statistics

Results are expressed as individual data points, mean ± standard deviation (SD). The Student *t*-test, paired *t*-test, Pearson’s correlation, or 1-way analysis of variance (ANOVA) with Bonferroni post hoc test was used for statistical analyses. *P* value < .05 was considered significant.
